# Mitogenomes of the four *Agathymus* holotypes collected 55 years ago

**DOI:** 10.1080/23802359.2017.1372701

**Published:** 2017-09-04

**Authors:** Jing Zhang, Qian Cong, Jinhui Shen, Nick V. Grishin

**Affiliations:** a Departments of Biophysics and Biochemistry, University of Texas Southwestern Medical Center, Dallas, TX, USA;; b Howard Hughes Medical Institute, University of Texas Southwestern Medical Center, Dallas, TX, USA

**Keywords:** Next-generation sequencing, phylogeny, primary type, biological collections, Megathymini

## Abstract

Giant-Skipper butterflies from the genus *Agathymus* (family Hesperiidae) are unusual as their caterpillars feed inside *Agave* leaves. Relationships among *Agathymus* taxa and their names (i.e. if they are species, subspecies, or synonyms) are poorly understood due to phenotypic similarity. DNA sequences are promising to clarify the taxonomic questions, but it is challenging to sequence name-bearing types that are usually old specimens with poorly preserved DNA. Using next generation sequencing, we assembled mitochondrial genomes of four *Agathymus mariae* group holotype specimens collected more than 55 years ago and housed pinned and dry in the American Museum of Natural History (New York, NY). We compared the holotype mitogenomes to those we obtained from fresh *A. mariae* specimens and the sister species *Agathymus micheneri*. All but *A. micheneri* mitogenomes were highly similar to each other (more than 99% identity), suggesting that the four names *chinatiensis*, *lajitaensis*, *rindgei*, and *gilberti* proposed by H. A. Freeman in 1964 may not refer to species-level taxa. The mitogenomes grouped eastern populations (*rindgei* and *gilberti*) together and apart from the western populations (nominal *mariae*, *chinatiensis*, and *lajitaensis*). Mexican *A. micheneri* differs by about 2.5% (about 5% in the COI barcode region) from *A. mariae*, and is likely to be a distinct species.

The Giant-Skippers (Hesperiidae: Megathymini) are endemics of the North American continent. They are known for their large size and unusual life habits. Adults do not feed and caterpillars live inside their foodplants: in *Yucca* roots and *Agave* leaves (Freeman 1969; Roever 1975; Scott 1986). Agave-feeding *Agathymus* is the most species-rich genus and its taxonomy is far from clear (Roever 1975; Scott 1986; Roever 1998). It remains to be studied which proposed names denote species, subspecies or synonyms. *Agathymus mariae* group includes skippers with caterpillars feeding on lechuguilla and distributed in western Texas, southern New Mexico and north-central Mexico (Freeman 1969). Six names exist for the populations in this group (Freeman 1969; Mielke 2005; Pelham 2008). In addition to nominal *A. mariae* (type locality: Texas, El Paso) (Barnes and Benjamin 1924) and Mexican *A. micheneri* (type locality: Mexico, Coahuila) (Stallings et al. 1961), four new taxa were proposed in 1964 as full species by H. A. Freeman (Freeman 1964). *Agathymus rindgei* and *A. gilberti* are sympatric with the same type locality (Texas, Kinney County, 14 mi north of Brackettville) and represent eastern populations of the *mariae* group. *Agathymus rindgei* and *A. gilberti* were suggested to have different number of chromosomes that correlated with the darker appearance and smaller yellow-orange spots of *A. gilberti*. *Agathymus chinatiensis* and *lajitaensis*, both from Presidio County in Texas, together with the nominal *A. mariae* constitute western populations and have more prominent pale spots on the hindwing below (Freeman 1969). As all *mariae* group taxa are morphologically similar, subsequent authors suggested that most, if not all, existing names correspond to either subspecies or synonyms (Roever 1975; Scott 1986; Pelham 2008). To further understand the relationships between these taxa, it would be instructive to investigate DNA differences between them.

Since the ultimate taxonomic study should be based on the analysis of name-bearing specimens, we sequenced, assembled and annotated the complete mitogenomes of the holotypes of Freeman's *A. mariae* group names. All four specimens have been housed pinned and dry in the American Museum of Natural History collection (New York, NY). Since the genitalia of all holotypes have been dissected, a small amount of tissue was sampled from the end of the abdomen through the cut introduced at the time of dissection. Methods for genomic DNA extraction, library construction, next-generation sequencing, and computational procedures have been reported by us previously (Shen et al. 2015; Cong et al. 2016a, 2016b; Shen et al. 2016; Zhang et al. 2017a, 2017b, 2017c). The mitogenome of *Agathymus mariae mariae* was used as a reference to search for (‘bait’) similar sequence reads using BWA (Li and Durbin 2009). The mitogenomes were assembled from these reads *de novo* using Platanus (Kajitani et al. 2014) followed by a manual gap-closing procedure. Due to possibly variable number of direct repeats that we previously found in the D-loop region (Zhang et al. 2017b), exact sequence of this region is uncertain in all Megathymini mitogenomes.

To assess the quality of the genomes of the holotypes collected 55 years ago, we sequenced a mitogenome of a fresh *A. mariae* group specimen from the eastern part of the range (voucher NVG-2413 from Texas: Val Verde County) and used the sequence we obtained previously (Zhang et al. 2017b) from a fresh *A. mariae* group specimen from the western part of the range. Finally, mitogenomes of two *Agathymus micheneri* specimens (one is a paratype) from Mexico: Coahuila, were sequenced using the same methods. Phylogenetic tree constructed from the mitogenomes reveals that *A. micheneri* is the most distant taxon from the rest, showing about 2.5% difference in mitogenome regions excluding D-loop and about 5% difference in the COI barcode region (Figure 1). Although originally proposed as a subspecies of *A. mariae*, *A. micheneri* is mostly treated as a full species in recent literature. Differences in its mitogenome support the status of *A. micheneri* as a species. Interestingly, the differences among mitogenomes of all other taxa are minimal: less than 0.6% in regions excluding D-loop and 0.7% in the COI barcode region, which is consistent with their possible conspecificity. Specimens group by locality rather than by age (i.e. fresh specimens are not clustered together in Figure 1), which supports sufficient quality of DNA sequences obtained from half-a-century-old specimens. Notably, specimens from the eastern (Kinney & Val Verde Counties) populations group together with 100 bootstrap support. Sympatric *A. gilberti* and *rindgei* did not reveal prominent differences in their mitogenomes, and divergence between them (0.44%) is not larger than that among named *A. mariae* subspecies (0.42–0.59%). As sympatric taxa cannot be subspecies of the same species, *A. gilberti* and *rindgei* are either synonymous, or mitochondrial introgression complicates the assessment, and the analysis of nuclear genes is necessary to fully resolve this taxonomic puzzle.

**Figure 1.**
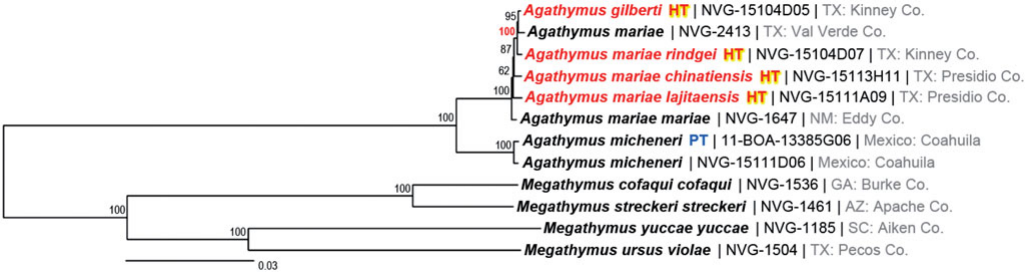
Maximum likelihood tree of mitogenomes of 12 Megathymini specimens. Specimen numbers are shown after the names followed by a general locality. Numbers by the nodes show bootstrap support values. GenBank accessions for sequences and data for *Agathymus* specimens are: *Agathymus gilberti* holotype, MF684854, voucher NVG-15104D05, female, USA: Texas, Kinney County, 14 mi N of Brackettville, elevation 1500′, 22 October 1961; *Agathymus mariae chinatiensis* holotype, MF684857, voucher NVG-15113H11, female, USA: Texas, Presidio County, 2.7 mi S of Shafter, elevation 4000′, 5 October 1960; *Agathymus mariae lajitaensis* holotype, MF684856, voucher NVG-15111A09, female, USA: Texas, Presidio County, 38 mi SE of Presidio, elevation 2650′, 2 October 1961; *Agathymus mariae mariae* KY630504, voucher NVG-1647, female, USA: New Mexico, Eddy County, 22-Sep-2013; *Agathymus mariae rindgei* holotype, MF684855, voucher NVG-15104D07, female, USA: Texas, Kinney County, 14 mi N of Brackettville, elevation 1508′, 23 October 1961; *Agathymus mariae* MF684859, voucher NVG-2413, female, USA: Texas, Val Verde County, SH163 S of Juno, 26 November 2013; *Agathymus micheneri* paratype, MF684860, voucher 11-BOA-13385G06, male, Mexico: Coahuila, 15–20 miles south of Allende, on highway 57, Km. 8, elevation 1300′, 2 October 1957; *Agathymus micheneri* MF684858, voucher NVG-15111D06, female, Mexico: Coahuila, 12 mi S Allende, 2 November 1964; *Megathymus cofaqui cofaqui* KY630503; *Megathymus streckeri streckeri* KY630501; *Megathymus ursus violae* KY630502; *Megathymus yuccae yuccae* KY630500.
